# A review of optical chemical structure recognition tools

**DOI:** 10.1186/s13321-020-00465-0

**Published:** 2020-10-07

**Authors:** Kohulan Rajan, Henning Otto Brinkhaus, Achim Zielesny, Christoph Steinbeck

**Affiliations:** 1 Institute for Inorganic and Analytical Chemistry, Friedrich-Schiller-University Jena, Lessingstr. 8, 07743 Jena, Germany; 2 Institute for Bioinformatics and Chemoinformatics, Westphalian University of Applied Sciences, August-Schmidt-Ring 10, 45665 Recklinghausen, Germany

**Keywords:** Optical chemical structure recognition, Named entity recognition, Data mining, Chemical data extraction, Chemical structure, Open data, Machine learning

## Abstract

Structural information about chemical compounds is typically conveyed as 2D images of molecular structures in scientific documents. Unfortunately, these depictions are not a machine-readable representation of the molecules. With a backlog of decades of chemical literature in printed form not properly represented in open-access databases, there is a high demand for the translation of graphical molecular depictions into machine-readable formats. This translation process is known as Optical Chemical Structure Recognition (OCSR). Today, we are looking back on nearly three decades of development in this demanding research field. Most OCSR methods follow a rule-based approach where the key step of vectorization of the depiction is followed by the interpretation of vectors and nodes as bonds and atoms. Opposed to that, some of the latest approaches are based on deep neural networks (DNN). This review provides an overview of all methods and tools that have been published in the field of OCSR. Additionally, a small benchmark study was performed with the available open-source OCSR tools in order to examine their performance.

## Introduction

A vast amount of knowledge is still hidden in the primary scientific literature, which is not accessible because the information is not properly curated and stored in open-access databases. Rediscovering this information and making it public is a complicated task that many data scientists took up as a challenge. This problem cannot be solved easily as the data available on chemistry and chemical structures are exponentially increasing by the day. While there is hope that the knowledge in future publications is readily deposited in a semantically well-annotated form in public archives, there is still a backlog of decades of chemical literature to be curated and stored in open-access databases.

In the synthetic sciences, natural products research, drug discovery and many other fields, there is a renewed interest to find more information about the known chemical structures and about the small molecules which are already published. Chemical knowledge that is collected in a time-consuming and expensive process often does not end up as structured information in databases. This way it remains inaccessible for re-use and databases may remain incomplete.

The literature can contain chemical information in various forms. Even though the textual information is presented in an unstructured manner, it does contain valuable information on the chemical compounds. In order to decode this information and create structured data, researchers developed chemical Named Entity Recognition (NER) systems [1]. On the other hand, decoding chemical structure information is a very different task with the goal of turning a graphical depiction into a machine-readable format. This research field is known as Optical Chemical Structure Recognition (OCSR).

The first work on OCSR was done in 1990 [2, 3] but the first complete system was published by McDaniel and Balmuth in 1992: Their program named Kekulé [4] planted the seed for this new field of research with a variety of commercial and noncommercial applications being released thereafter. The first systems followed similar approaches in handling the chemical structure images, but differed in implementation details and the accuracy of rebuilding the chemical structure from an image. They shared rule-based heuristics for treating a segmented image of a single chemical structure and methods like image vectorization, image disassembly into separated components, image thinning, enhancement of the line, resolution of components using Optical Character Recognition (OCR), and eventually the reconstruction of the graph representation of the molecule. Unfortunately most of the commercial OCSR systems were inaccessible to academic researchers.

Closed-source systems were very popular in the early 1990s. The first complete open-source system called Optical Structure Recognition Application (OSRA) [5] was published by Filippov and Nicklaus in 2009. OSRA helped many early-stage researchers in drug development and natural product research. Its success as an open-source tool allowed the development of subsequent open-source systems such as Imago [5, 6] and the recent Java-based tool MolVec [7].

Recent developments in hardware and Deep Neural Networks (DNNs) in machine learning led to outstanding achievements in image recognition technologies [8]. With the cost of the hardware for machine learning getting lower and the accessibility of open machine learning libraries such as TensorFlow [9], Pytorch [10], and Caffe [11], barriers to the implementation of machine learning-based chemical image recognition systems were lowered.

In this review, we have analyzed all the available optical chemical structure recognition systems published. We found only three open-source systems available while the rest of them are commercial tools. Some publications describe methods that were developed without any working system ever being published. We discuss the algorithms behind these systems, their architectures, and how well they perform in real-world situations. For the available open-source systems, we carried out a small benchmark study and the results are reported here.

## A general overview of the systems

Nowadays, the chemical structure depictions published in the literature are generally submitted to journals as raster images. Older publications often contain whole scanned pages from the original printed literature. In general, when a chemical structure is drawn using common structure editors, the file formats are easily interconvertible with other machine-readable formats such as SMILES [12], connection tables or SDfiles [13]. Once the depiction is saved as an image, it is very difficult to decode it back into a machine-readable representation of the molecule. In order to automatically feed and maintain publicly available databases with information about molecules, the reliable retranslation of the image of a chemical structure depiction is required.

Ideally, an OCSR system should be able to detect a chemical structure printed in the literature and segment the structures out of the whole page which also contains other graphical elements and text. This segmented structure should then undergo a preprocessing step such as denoising the image to get rid of unwanted pixel information and binarization to remove all the RGB (Red, Green, and Blue) values that are given to each pixel when they are produced. Once the preprocessing is done, the OCSR system should extract all relevant features from the input image and later use this extracted information to build up a meaningful chemical structure.

To do this, most OCSR systems follow a common method, where the image is vectorized and segmented into separate elements. The atom information, printed with atom symbols, will be recognized using OCR, while the bond information for single, double, triple, edged, dashed, or dotted bonds is stored in the lines of the depiction which is difficult to interpret. For this purpose, the OCSR tools use a line detection algorithm such as a Hough transform [14]. The detected line information is then analyzed thoroughly to recognize the different line types due to line length, width, spacing, thickness, and arrangement. With this information, all bonds are identified. Afterwards, a separate algorithm is used to detect the positioning of the atom characters within the bonds. Finally, with all the detected information, an algorithm rebuilds the complete molecular graph which is post-processed to get a semantically valid molecule. Once the post-process step is over, the output is generated as a SMILES string, a connection table, or as an SDfile.

Most of these tools cannot handle a whole scanned literature page, they usually need an input of an image with a single chemical structure. Only a few tools like OSRA, Imago, and CLiDE can be used for complete page recognition. As opposed to the rule-based methods, a few machine learning-based methods have been developed: These tools completely rely on the recognition of chemical structures without any hardcoded rules.

Table 1 summarizes information about the available tools and methods as well as their differences. In addition, each approach is reviewed separately for a better understanding.

**Table 1:** Comparison of tools and methods published

Tool name	Programming language used	Operating System compatibility	Open-source	Commercial or free availability (2020)	Ongoing development
Kekulé	C + +	Windows	No	Yes	No
OROCS	C	IBM OS/2	No	No	No
CLiDE Pro	C + +	Windows	No	Yes	Yes
OSRA	C + +	Independent	Yes^a^	Yes	Yes
ChemReader	C + +	Windows	No	No	No
MolRec	Unknown	Unknown	No	No	Unknown
Imago	C + +	Independent	Yes	Yes	No
ChemOCR	Java	Independent	No	Yes	Yes
ChemInfty	Unknown	Windows	No	No	No
eChem	Unknown	Unknown	No	No	No
MLOCSR	Unknown	Only Web interface	No	Only web interface	Unknown
OCSR	Unknown	Unknown	No	No	Unknown
ChemRobot	Unknown	Unknown	No	No	Unknown
MolVec	Java	Independent	Yes	Yes	Yes
MSE-DUDL	Python	Independent	No	No	No
Chemgrapher	Python	Independent	No	No	Yes

^a^Precompiled tool is only available commercially

## Rule-based systems

### Kekulé

In 1992, one of the first complete working OCSR tools, *Kekulé* [4], was released. Kekulé uses a scanned image provided by a user and vectorizes it. The binary image of this chemical structure diagram is used for resolving the characters and lines. Kekulé applies a rule-based approach to generate a connection table and has a graphical user interface that enables the inspection and editing of the results.

The workflow consists of scanning, vectorization, search for dashed and wedged lines, optical character recognition, graph compilation, and post-processing. After the final postprocessing step it displays the results and allows for editing. During the scanning step, the area on a page that contains the structure diagram is selected and segmented into a separate TIFF image. Then, the image is thinned, vectorized, and smoothed. Dashed and wedged lines are identified and treated as connected elements. For OCR, a multilayer perceptron neural network is used after the application of a preprocessing procedure for normalization to achieve results with 96% accuracy. The resolved characters are corrected with a set of rules and typical spelling mistakes (e.g. ‘S’ vs. ‘5′). The resulting strings that contain only one character are subsequently merged based on their relative position. For the graph compilation, all character positions are treated as nodes, and all dashed/wedged bonds are treated as edges. Then, these elements are removed from the image and the remaining molecule skeleton is used for further steps. Each remaining vector is assumed to represent a bond and each point between two vectors is treated as a new node unless there already is a node due to the presence of an OCR result. The width of the lines that represent the vectors in the original image is analyzed in order to recognize stereo information. If multiple vectors connect to the same nodes, the bond order between these nodes is increased accordingly. Finally, all the gathered information is combined into a single graph. In a post-processing procedure, superatom labels are resolved (e.g. “Ph” to a phenyl group). Additionally, the user is asked to provide the label wherever the OCR failed. Circles are detected and translated into alternating single and double bonds and all bond crossings are evaluated to determine whether they represent a node or not. The final results are displayed in the GUI and the graphical output can be adjusted further by a user.

### Optical recognition of chemical graphics (OROCS)

In 1993, a publication about an OCSR tool by IBM was released [15]. The workflow consists of nine steps: scanning, separation, vectorization, segmentation, cleanup, OCR, structure recognition, aggregation, and post-processing. For the scanning of an analog image, a resolution of 300 dpi is necessary. The image is then divided by defining polygon-shaped bounding boxes around all connected elements. Then, the program searches for all components that are bigger than a threshold that is defined according to the maximum character size on the page. Sufficiently close components are allocated to one another and their bounding boxes are merged. The part of the image that is framed by the resulting bounding box is considered to be a chemical structure. After a vectorization step, each vector is classified as belonging to a character, the bond structure, or other elements that constitute a chemical structure diagram according to a set of rules. The vectors that describe the structure itself are cleaned up automatically for certain cases (e.g. for the case that a straight line has been interpreted as two vectors). Additionally, a user interface enables correcting vectors manually. Subsequently, the molecule graph is generated using the information from the vector image. During this process, each vector is interpreted as a bond and each connection point is interpreted as an atom. If there is no other label, an atom is assumed to be a carbon atom. In order to interpret the remaining labels, the coordinates of the vectors that were allocated to characters in the original raster image are used for the feature-based OCR. The resolved labels are then translated into the corresponding substructures. If a text element is not at a node position, it is considered irrelevant and is deleted. After replacing every detected circle with alternating single and double bonds, the creation of the connection table is complete. Finally, the validity of the connection table is verified and it can be manually edited in a user interface.

### CLiDE

Another OCSR software solution named **C**hemical **Li**terature **D**ata **E**xtraction (CLiDE) [16] was released in 1993. This consisted of a workflow with three phases: the recognition phase, the text grouping phase, and the interpretation phase.

During the recognition phase, the outer contours of each connected element are determined and approximated with a polygon. The largest character size is estimated based on the distribution of the sizes of the elements. Each element is then classified as a character, a graphic, or as a dash based on size and relative height. The graphic primitives (lines) are recognized by searching for two long parallel edges in a polygon with short sides at the end that can be approximated as a curve. Eventually each primitive is saved as the start and end points of the two long sides of the corresponding polygon. There is a second routine for the identification of dashed bonds. The relative position of connected elements that have been classified as dashes due to their size is evaluated. If the dash objects are aligned on a straight line, the line and a single coordinate for each dash are saved.

The OCR is performed with a neural network that has been trained for this purpose. Words on a page are then grouped based on their relative position to determine lines and text blocks. This comprehensive OCR system which is technically capable of resolving the text blocks has not been included in the initial software package. Only the text elements which are associated with chemical structures are used for further processing.

The interpretation phase is then used to generate the connection table of the molecule based on the previously identified graphic primitives and the resolved text elements. Atom and superatom labels are resolved using an internal database. The final result can be saved in a molfile [13].

### Further development of CLiDE

CLiDE Pro [17] is a commercial OCSR tool. The workflow begins with the identification of chemical images. Therefore, the input image (of a page) is binarized and subdivided into connected elements that are described by their contours. Elements like words are grouped into lines and text blocks, depending on their relative position. Other elements are considered to be part of the graphics blocks.

The second phase is the generation of a connection table that represents the molecule. First, the connected compounds in the graphics blocks are classified as characters, dashes, lines, graphics, and noise. The classification is done according to the relative size of the connected elements and a row of features, like the pixel density and the number of contours. Then, all elements that have been classified as lines and graphics are vectorized by approximating the contours with a polygon. Ideally, a simple line is represented as two long straight sides with a couple of short sides at the ends that can be approximated as a curve. Each vector in the image is described by the start and end points of the two long borders that constitute the polygon. Dashed bonds are identified differently. If elements which have been previously classified as dashed can be arranged on a line, they are considered to represent a dashed bond. Dashes that cannot be allocated to a dashed bond are reclassified as characters. For the construction of atom labels, an OCR engine is applied to resolve all text character by character. These characters are then grouped into words based on their relative position. The resolution of the identified labels is done using an internal database that contains atom and superatom labels and their machine-readable representations. This information is combined with the previously analyzed graphical elements that constitute the structure diagram to generate the connection table of the molecule.

Chemical structure depictions commonly contain R-group labels. This means that the depiction contains a variable (often represented by the character ‘R’) which refers to a structural element. The allocation of one or more structural elements to the variable is usually defined below the structure. CLiDE Pro is capable of processing common R-group notation styles. Therefore, text blocks that describe the value of R-group variables are identified. Each text block is then allocated to a structure diagram based on proximity and the amount and type of R-group variables.

The article about CLiDE Pro was published with a test set of 454 images which contain 519 chemical structure diagrams [17]

### OSRA

With the publication of OSRA [5] in 2009, the first open-source OCSR tool was available. Since its original release, OSRA has been continuously improved and refined. Like all other tools that were mentioned before, it follows a rule-based approach: First, the input image is converted to grayscale and binarized. Rectangular areas containing chemical structures are defined by their dimensions and the ratio of black and white pixels in the selected area. If a segmented rectangle is found to contain a lot of noise, noise removal and anisotropic smoothing are performed. Subsequently, a thinning algorithm is applied before vectorizing the image. Based on a set of rules, control points and vectors are interpreted as atoms and bonds. Atom labels are identified using two OCR systems. Then, aromatic, double, and triple bonds are recognized as circles, or parallel lines that have the average bond length. If the development of the thickness of an object can be approximated by linear regression, it is interpreted as a dashed bond. Multiple small objects within the average bond length are interpreted as a dashed bond. Finally, the connection table of the molecule is generated by combining the information that was gathered in previous steps.

For the resolution of the atom and superatom labels, OSRA uses a dictionary of the labels (and different spelling varieties) which can be modified by the user. A superatom label describes a structural element such as ‘MeO’ for a methoxy group. Polymer structures, as well as reactions, can be recognized. By default, every input image is processed at three resolutions. OSRA then applies an empirically determined confidence estimation function and keeps the result with the best confidence value only [5, 18]. OSRA can be used as a command-line tool on a local machine and an online application.

Due to OSRA being an open-source tool, other developers were able to implement it in their projects and to develop improvements by using it in the backend. ChemEx is a tool that combines OSRA in combination with a text mining workflow in order to mine natural-product-related data from scientific publications [19]. In 2020, the tool ChemSchematicResolver was published with Python bindings for OSRA (PyOSRA). ChemSchematicResolver implements a segmentation algorithm in combination with OCR to allocate the resolved chemical structure diagrams to their names and labels. It then combines the OCSR results with information that was gained with the text mining tool ChemDataExtractor [20]. The capability of reading labels is also used in PyOSRA to resolve common R-group notations and implement this information in the resolved chemical structure diagram.

### ChemReader

ChemReader [21] was published in 2009. The workflow begins with a preprocessing step involving noise removal and size normalization. Subsequently, characters and lines are separated based on their height, area and relative position to other characters. After the detection of lines using an adapted Hough transform, the bond order and stereochemical information are determined. There is an additional routine for the detection of pentagonal and hexagonal structures within the molecule. Additionally, circles within cyclic structures in the molecule are detected. The previously identified characters are resolved using OCR. The resulting labels often contain errors and are corrected in a consecutive spelling correction step using a dictionary and a set of rules about valid valence. Different candidates are considered for each character and evaluated according to a confidence score. Finally, a graph representation of the molecule is compiled based on the previously detected features. Even though the authors announced in 2009 that ChemReader would become available as a commercial tool, it has not been published yet.

### MolRec

In 2009, a researcher from the University of Birmingham in the United Kingdom announced that he was working on an OCSR tool [22] that he intended to release openly available once it would reach a sufficient standard. The proposed application mainly uses a combination of the known techniques which are already in use. It has a total of 6 steps. Image binarization is done using Otsu’s method [23]. All connected components in the image are defined using the grass-fire algorithm [24]. In the next step, these components are processed and the characters are separated from other (graphical) elements. Wedged bonds and the dashed bonds are recognized separately using a set of rules. All remaining elements are thinned using Hilditch’s algorithm [25]. Then, all line characters, their connections, and endpoints are interpreted as the bond structure of the molecule. Finally, the extracted information about the given molecule is combined in a graph representation. Based on this graph, a molecular formula and a SMILES string are created.

Three years later, a publication about the OCSR system MolRec [26] was released. Here, the workflow also begins with binarization, detection of connected components, and resolution of characters as well as their subsequent removal from the image. After a thinning step, the resulting set of lines and polylines is processed using the Douglas-Peucker line simplification [27]. MolRec works with the assumption that there is a limited set of geometrical primitives that assemble a chemical structure diagram (line segments, arrows, circles, triangles, and characters). It then applies a sophisticated set of rules in order to interpret these geometrical primitives as a part of a molecule. Superatom labels are resolved using a dictionary. The gathered information is compiled in a graph representation of the molecule. To our knowledge, MolRec is neither freely available nor as a commercial tool.

### Imago

In 2011, a second open-source OCSR tool was published—Imago [5, 6]. As the other tools, it implements a similar rule-based approach: The input image is blurred, binarized, and segmented into connected items. Dashed bonds are recognized separately and removed from the image for further processing. Then, the remaining objects are allocated to a *symbols layer* which contains the atom and superatom labels and a *graphics layer* that contains the bonds. This classification is based on the width to height ratio of the elements and a set of rules. The elements in the graphics layer are processed to create the graph representation of the molecule. Therefore, the graphics elements are thinned, junctions are removed, a smoothing algorithm is applied and the image is vectorized. After the merging of close nodes, all edges are interpreted as bonds. Parallel lines are detected and the bond order is adapted correspondingly. All labels from the symbols layer are resolved and linked to the nodes, and the stereo information from the previously removed dashed bonds is added. Imago uses a dictionary of superatoms and common abbreviations. It can be used from a user interface or as a command-line tool for batches of images.

### chemOCR

chemOCR [28] is an extension of the work done by Mark Zimmerman and Maria-Elena Algorri [29, 30] as it uses the earlier established method from these publications. The method uses its own pattern recognition algorithms combined with a support vector machine (SVM) to detect and interpret an image containing a chemical structure.

chemOCR is written in Java. At the backend, it uses a set of algorithms that were previously developed by the same authors and a set of rules which they propose to process any type of image regardless of its drawing type.

chemOCR has multiple modules incorporated into an overall OCSR workflow. The tool accepts a PDF document or an image that consists of chemical structures. PDF documents are converted into separate bitmap images and processed further. The images which contain structures and other information such as text blocks, tables, and reactions will undergo a preprocessing step where all components not related to chemical structures are removed from the image. The authors claim that the tool works better with images that only contain the chemical structure. In the next steps, images are enhanced, the connected components with the connecting text areas are identified, an OCR module detects the characters attached to the structure, and the chiral bonds which are represented by wedged bonds are detected. In the final preprocessing step, the image is converted into a set of vectors.

The vectorized image with the detected components is then used in the reconstruction of the chemical structure. This procedure is divided into two main tasks. First, an expert system analyzes all the information from the preprocessing steps, using an algorithm that determines the orientation of the graph and annotates the connected components. The second task is the assembly of the molecule based on the annotated components and a set of rules in a stepwise manner until the complete structure is reconstructed.

All reconstructed chemical structures will be further processed by a post-processing step where the fragments of the chemical structures will get split, saved as separate structures in an SDfile and the final set of results will be displayed. Later on, a validation step is carried out on the reconstructed chemical structures to give an overall confidence score on the final results. According to the developers, the tool has a 65.6% accuracy (656 structures were correctly reconstructed out of 1000 unique images) and is currently available as commercial software.

### ChemInfty

ChemInfty [31] was developed as a robust solution to resolve chemical structures in Japanese patents. The input image is binarized and smoothed. Additionally, text captions are removed. Characters are resolved using a custom OCR engine. If characters are resolved with a high confidence level, they are removed from the image. Then, the image is thinned and crossing points and bending points are identified. The remaining elements are divided into lines and curves. After a bond recognition step, the elements are grouped and the “most suitable combination” is determined. Then, the remaining characters are resolved by using a custom OCR engine. According to the authors, ChemInfty has the advantage of dealing with characters in labels that touch the lines in the structure diagram.

### eChem

eChem [32] was developed as a helper tool for chemistry students. The main goal of the application is to help students to understand chemical structures and how to use them in reactions.

The OCSR in this application is used as a module, which processes a chemical structure and the data generated from this module will be forwarded to other modules for further processing.

The application comes with multiple modules for the user to use. The user can either upload a soft copy of a chemical structure or a scanned copy, this will be processed by the chemical structure name recognizer module of the system to translate the raster image into a computer-readable file format. The transformation of images to a computer-readable file format is somewhat similar to the work done by Mark Zimmerman in 2007. The input images are resized and denoised to set an optimal value of the resolution. They get segmented into separate components based on their pixel connectivity. The separated characters rather than bonds are detected using the Microsoft Office Document Image Library. Then, the bonds (lines) are detected using a modified version of the Hough transform. The detected bonds are separated into single, double, or triple bonds depending on their overall thickness. The recognized characters combined with the recognized structure are used to generate a SMILES string. Invalid SMILES strings are recognized in a spell checker algorithm before the final output is generated.

This application further has a component to help the user with generating chemical reactions from the detected structures. In order to use this component, the user needs to define all the reactants used in the desired reaction. The reaction component follows a knowledge base that is predefined to carry on with the reaction steps which helps to generate the final output.

The authors didn’t state any availability of this method as open-source software.

### Markov logic networks for OCSR

MLOCSR [33] is an OCSR method that follows a pipelined design strategy which is a combination of low level and high-level processing. The workflow is divided into three modules. The first module is a low-level extractor that extracts the graphical entities and text elements. Then, a high-level module uses a Markov Logic network [34, 35] in order to clean up the noisy low-level data and to add more information using a knowledge base. The last module processes the previously produced information and assembles a graph that represents the depicted molecule.

The MLOCSR workflow begins with the preprocessing of the image in order to extract low-level entities like graphical primitives and text elements. Therefore, the input raster image is binarized and a smoothing algorithm is applied. Then, the bounding boxes of all connected components are determined. Subsequently, textual and graphical elements are separated. Based on the identified text elements, the text height is estimated. This information is used to filter the input for the OCR engine. After the removal of text elements, the remaining image is vectorized by applying a contour-based technique, and different kinds of lines (e.g. dashed or wavy lines) are classified.

The extracted graphical primitives need to be interpreted in order to assemble a connection table that represents the molecule that is depicted in the original chemical structure diagram. Since the information extracted by the low-level module is noisy, it needs to be modified based on more information about the composition of atoms and bonds in a molecule. Due to the complexity of this problem and the clear rules defining the valid assembly of a molecule, a Markov logic network is used to assign probabilities to mappings of elements that result in a representation of a molecule. The rules which are incorporated in the Markov logic network are saved as a knowledge base.

Finally, using the resulted output from the Markov logic network, the entities are reassembled to form a valid chemical structure. This structure is first generated as a connection table and then converted into a molfile. The tool is not distributed as an open-source software but a web version of it is available for the public.

### OCSR

Another OCSR tool (name: *OCSR*) [36] was proposed in 2015. The workflow begins with a grayscale conversion and binarization of the input image. Characters are resolved using OCR. A procedure for the detection of wedge bonds is implemented. A thinning algorithm is applied and the image is vectorized. Recognized characters are merged to form atom and superatom labels. The gathered information is combined in an adjacency matrix or a string representation of the molecule. To our knowledge, this tool is not available.

### Chemrobot

In 2017, a US patent for the OCSR tool Chemrobot [37] was filed. The workflow begins with the conversion to grayscale, a binarization and a smoothing step of the input image. Circles within cyclic structures in the chemical structure diagram are recognized as aromatic bonds and OCR is used to identify characters. A thinning algorithm is applied and edges of the molecular graph are detected. After the detection of double and triple bonds, the result is compiled in common output formats. The authors claim that the tool is suitable for the interpretation of hand-drawn structures from noisy input images.

### MolVec

MolVec [7] is an open-source Java-based tool developed by a group of scientists from NIH. The tool is an attempt to solve the common problem researchers were having using other public tools. MolVec attempts to simplify its use by being a self-contained, lightweight standalone tool that does not require any programming skills. MolVec is completely programmed in Java, it needs JAVA 8 or higher to run locally. It can be used as a command-line tool, through the Java API, a user interface or online [7]. As it is open-source software, developers who would like to modify the tool can access it on GitHub. It currently only accepts images with a single chemical structure, but it can handle any type of image resolution. Further development to recognize separate structures is currently ongoing work. So far, the researchers have not published a scientific paper about their work so that there is not much information about the algorithms running behind the scenes. According to the developers, the algorithm consists of binarization, line thinning and shape, feature, node and edge detection. The detected elements are adjusted and assembled based on a set of rules.

## Machine-learning-based systems

Two new deep learning methods in OCSR have been implemented recently. In 2003, Gkoutos et al. [38] used a Kohonen network [39] in the backend to distinguish chemical structure images from non-chemical images such as photographs. Additionally, a support vector machine (SVM) [40] based classification is included in ChemOCR. A complete machine learning-based OCSR method has not been published until 2019. In the following section, the two published methods which use deep learning for OCSR purposes are summarized. Since they are closed-source systems, neither of them was available for testing.

### MSE-DUDL

In 2019, Staker et al. [41] presented a data-driven, deep learning based approach for OCSR called Molecular Structure Extraction from Documents Using Deep Learning (MSE-DUDL). The system uses two types of networks in the backend: a segmentation network and a structure prediction network. The segmentation network is used to scan through the images containing chemical graphs and other elements such as text blocks, tables, reactions. It then identifies the chemical structure diagrams and segments them out from the images. This network follows a convolutional neural network (CNN) architecture based on an open-source implementation of *U-Net* which was used by Ronneberger et al. [42] for their work on biomedical image segmentation. This architecture has the ability to support full-resolution detection and a fine-grained segmentation. The network has a contacting path where the input gets transformed (downsampling) into a latent representation, and an expansive path where the latent representation is expanded (upsampling) until it matches the resolution of the input image. The final predicted output is used to obtain pixel masks which helps the network to do the extraction of the images. The network was trained on a manually curated set of images that were extracted from documents and edited. The model had 380,000 parameters and was trained for 4 days on a single graphics processor unit (GPU).

The prediction network follows an encoder-decoder architecture where the encoder encodes the images containing chemical graphs to a fixed-length latent space using a CNN and then the decoder uses a recurrent neural network (RNN) to decode them back to a sequence of SMILES characters. The CNN is architecturally similar to the ImageNet [43] architecture and the RNN follows the sequence to sequence learning network [44] used for the English to French translation model. The model was trained on 57 million images generated using the Indigo toolkit [45] with molecules retrieved from PubChem [46] and on another dataset of 1.7 million images generated by Indigo using molecules retrieved from the publicly available data from the United States Patent and Trademark Office (USPTO) [47, 48]. This model had 46.3 million parameters and training took 26 days on eight GPUs.

After training the networks, the authors tested the models using a validation dataset which is 10% of the Pubchem dataset and 25% of the USPTO dataset, and they observed a high accuracy on both datasets. The whole system works well in a complete end-to-end fashion with reasonable accuracy as stated by the authors. Unfortunately, the method is not available as an open-source tool.

### Chemgrapher

Chemgrapher [49] is a deep-learning OCSR method implemented in a modular style. It analyzes a given image of a chemical structure in order to rebuild it as a computer-readable graph representation. The main motivation behind this method is to have a deep learning model for optical compound recognition. Primarily, the model can be divided into two sub-sections. The first section deals with segmentation and the second section deals with the location recognition for atoms, bonds and charges, with the resulting output being processed by a separate algorithm to build the chemical graph. All these networks are based on CNNs. The segmentation networks follow the idea of dilated convolution described by Yu and Koltun [50].

The segmentation network was trained on chemical structure images generated with RDKit [51] based on data retrieved from CHEMBL [52]. The output of the segmentation network is used as an input for the classification networks to locate atoms, bonds and charges.

Every network was checked individually for accuracy and it is stated that the classification networks perform much better than the segmentation networks. The overall accuracy of the model is determined by the resulting chemical graphs. According to the authors, the system outperforms OSRA.

## Comparison of the open-source OCSR tools

### Materials and methods

In order to compare the results of the three available open-source OCSR tools Imago (version 2.0), MolVec (version 0.9.7) and OSRA (version 2.1.0), multiple datasets which are freely available online were analyzed according to the validation procedure of the OSRA developers [53]. The datasets were:

1. A set of 5719 images of chemical structures and the corresponding molfiles (based on data from the USPTO) obtained from the OSRA online presence [53].
2. The dataset (UOB) of 5740 images and molfiles of chemical structures developed by the University of Birmingham, United Kingdom, and published alongside MolRec [54].
3. The Conference and Labs of the Evaluation Forum (CLEF) test set of 961 images and molfiles published in 2012 [55].
4. A subset (450 images and SDfiles) of a dataset published with ChemInfty (see above) based on data from the Japanese Patent Office (JPO), obtained from the OSRA online presence [53]. (Note that this dataset contains many labels (sometimes with Japanese characters) and irregular features, such as variations in the line thickness. Additionally, some images have a poor quality and contain a lot of noise.)

The TIFF images were converted to PNG images with a resolution of 72 dpi to assure comparability, as MolVec and Imago both showed problems handling those TIFF files in batch mode.

The command-line version of Imago [56] was executed without installation by running the following command in the directory with the executable file:
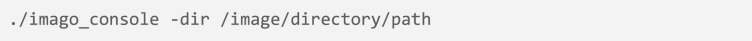


MolVec was downloaded as a jar file with all of its dependencies. It was executed from the command-line by running:
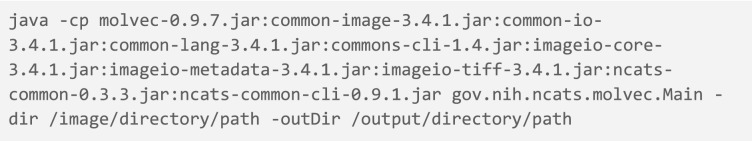


OSRA was installed in an Anaconda environment using the Conda recipe for PyOSRA which was published by Ed Beard [57]. This was done analogously to the installation instructions in the ChemSchematicResover documentation [58]. We used the PyOSRA environment because compiling OSRA from source code is excessively complex as it has a lot of dependencies that need to be compiled from their own source code as well. There is the option to obtain a commercial license to get a precompiled version of the software.

OSRA was then executed on the test data set by running the following shell command in the directory with the images. Here, it is necessary to specify the location of the dictionaries for superatoms and common spelling corrections.
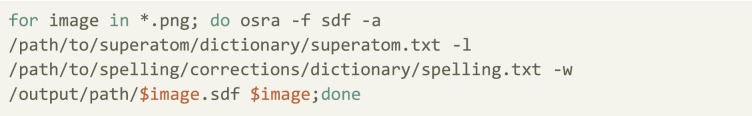


The accuracies of the tools listed in Table 2 below correspond to perfectly recognized structures according to a perfect match of the Standard InChI strings [59] that were created based on the OCSR results and the reference files.

**Table 2:** Time elapsed and accuracy reported for the open-source OCSR tools

Dataset		MolVec 0.9.7	Imago 2.0	OSRA 2.1
USPTO (5719 images)	Time (min)	28.65	72.83	145.04
	Accuracy	88.41%	87.20%	87.69%
UOB (5740 images)	Time (min)	28.42	152.52	125.78
	Accuracy	88.39%	63.54%	86.50%
CLEF 2012 (961 images)	Time (min)	4.41	16.03	21.33
	Accuracy	80.96%	65.45%	94.90%
JPO (450 images)	Time (min)	7.50	22.55	16.68
	Accuracy	66.67%	40.00%	57.78%

All the processing was done on a Linux workstation running with Ubuntu 20.04 LTS, which has 2 Intel(R) Xeon(R) Silver 4114 CPUs capable of handling 40 threads and with 64 GB of RAM.

## Results

**Fig. 1:**
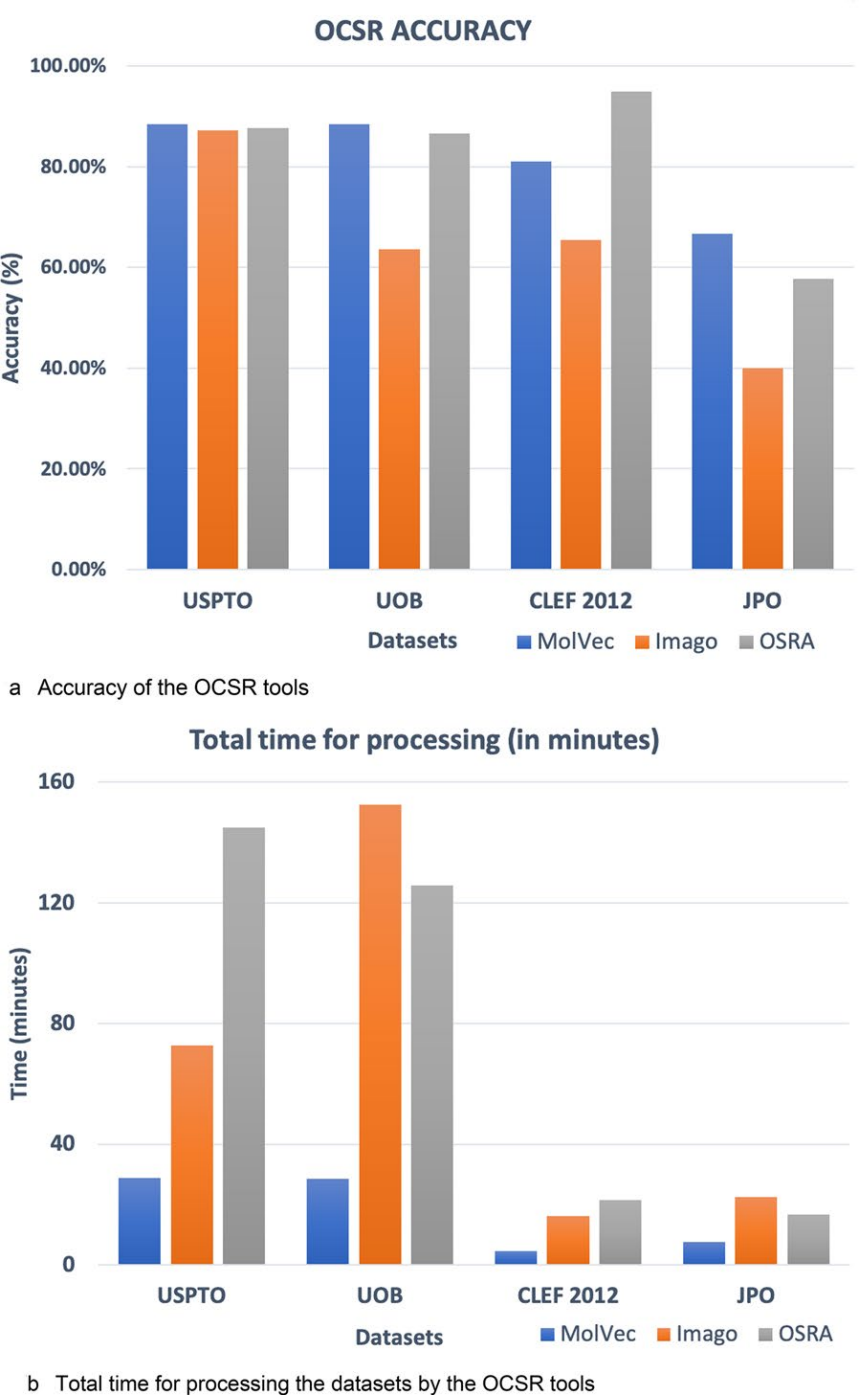
**a** Accuracy (Right: higher the better) and **b** Total time for processing (Left: lower the better)

As shown in Table 2, MolVec processes the images significantly faster than its competitors. All three tools performed fairly well on the given set of images. As illustrated in Fig. 1, the proportion of accurate results produced by MolVec and OSRA with the UOB, CLEF and JPO datasets was approximately 20% higher than in the results produced by Imago. The lower overall performance of all three tools with the JPO dataset is likely due to the lower quality of the depictions, the presence of labels and other irregular features. The extraordinarily good performance of OSRA on the CLEF dataset is a notable observation. The examination of the images in the dataset reveals a set of well-segmented, clean chemical structure depictions which is seemingly handled especially well by OSRA.

## Discussion

In the years 1992–1993, the first developments in the field of OCSR were reported. These early developments were commercial, but there have been open solutions since 2009. In this review, we analyzed all freely available tools used in OCSR. Additionally, we summarized the methods which have been described but never became available as testable software. There are different OCSR tools being used in industry and the gold standard has never been set.

Out of all the tools reviewed here, we only used the three freely available open-source tools for our benchmark study to see how well they perform, how fast they are, and how accurately they recognize the chemical structure. The results indicate that all three tools perform well on the USPTO dataset. MolVec is significantly faster than the other two tools and has the additional feature of a pre-implemented parallelization function. For the OCSR-assisted manual extraction from documents, Imago offers a user interface that enables the selection of the desired segment which contains a structure. OSRA gives the user a wide variety of adaptable options in order to optimize the extraction for custom purposes. For the processing of documents, it has a segmentation algorithm without a user interface. MolVec has a user interface without a page reader but the developers are working on integrating this function in future versions.

Most of the discussed methods are not implemented in any available tools. They are simply methods describing a certain algorithm the authors developed, or in some research articles they are presented as a prototype, but the corresponding tools were never published. In practice, this is a huge lost potential in this field as researchers are not able to access the implementation of thoroughly planned and executed ideas.

The majority of the tools follow a common rule-based approach which is based on the interpretation of the elements in the vectorized image as nodes and edges in a graph representation of the molecule. Many tools seem to mirror each other's ideas and add some modifications in the algorithms while the basic workflow mostly remains the same. Only two OCSR methods address the implementation of deep neural networks as the backbone of their tool and neither of them is not openly available.

The recently published ChemSchematicResolver combines text mining with OCSR in order to retrieve all the information possible from the printed literature. A major problem in the field of automated generation and curation of databases is the missing linkage of the information mined by OCSR tools to the corresponding names. To our knowledge, ChemSchematicResolver is the first tool that addresses this problem to enable the mining of information on a large scale and in an unsupervised manner. As it uses a Python implementation of OSRA, this development also represents a good example for constructive synergies in the open-source community.

We could see that none of the openly available tools works perfectly. This may make things difficult for researchers when the combination of multiple tools is necessary for a better overall result. There are no tools available having all of the following features—OCR based complete page reader, image segmentation, batch processing, and natural language processing (NLP). Additionally, the interpretation of R-group labels remains an unsolved problem when the allocation of structural elements to the R-group variables is presented elsewhere in the text or in tables.

There is a need for a completely automated, feature-rich, reliable OCSR application that can process complete documents in order to directly create database entries and it should allow a user to cross-reference the mined information with the already existing data.

## Conclusion

The move to open-access, high-quality scientific information systems creates a demand for automatic curation of knowledge from the printed scientific literature. In chemistry, this includes the translation of images of chemical structures into a machine-readable format, which is one of many steps towards the development of more complete curation systems.

Within the last decade we have observed increased activity in this field. We see that rule-based systems were mostly developed, but there were also two deep learning based solutions. The first ever open-source tool OSRA was released in 2009 and it is still being developed. There are two alternative open-source tools apart from OSRA, Imago and MolVec. Our examination of the performance of the three open-source tools showed an average accuracy of above 80% for OSRA and MolVec which can be considered acceptable. Nevertheless, there is potential for further improvements.

This review is an attempt to give an overview of three decades of research in the field of OCSR. We have discussed the development and improvements of methods that allow the automated extraction of chemical information from the literature. In particular, the freely available tools open up opportunities for the combination of OCSR tools with text mining to achieve the complete automated extraction of chemical information from the literature.

## Data Availability

The modified PNG images used in the comparison of the open-source toolkits are available at: https://github.com/Kohulan/OCSR_Review

## Abbreviations

CLiDE: Chemical literature data extraction
CNN: Convolutional neural network
CLEF: Conference and labs of the evaluation forums
DNN: Deep neural network
GPU: Graphics processor unit
GUI: Graphical user interface
JPEG: Joint photographic experts group
JPO: Japanese Patent Office
MLOCSR: Markov logic networks for optical chemical structure recognition
MSE-DUDL: Molecular structure extraction from documents using deep learning
NER: Named entity recognition
NLP: Natural language processing
NIH: National Institute of Health
NN: Neural network
OCR: Optical character recognition
OCSR: Optical chemical structure recognition
OROCS: Optical recognition of chemical graphs
OSRA: Optical structure recognition application
PDF: Portable document format
PNG: Portable network graphics
RNN: Recurrent neural network
SDFile: Structure-data file
SMILES: Simplified molecular-input line-entry system
SVM: Support vector machine
TIFF: Tagged image file format
UOB: University of Birmingham, United Kingdom (dataset)
USPTO: United States Patent and Trademark Office
